# Formation of active inclusion bodies induced by hydrophobic self-assembling peptide GFIL8

**DOI:** 10.1186/s12934-015-0270-0

**Published:** 2015-06-16

**Authors:** Xu Wang, Bihong Zhou, Weike Hu, Qing Zhao, Zhanglin Lin

**Affiliations:** Department of Chemical Engineering, Tsinghua University, One Tsinghua Garden Road, Beijing, 100084 China

**Keywords:** Active inclusion bodies, Hydrophobic self-assembling peptide, Intein-mediated cleavage, Expression and purification coupled tag

## Abstract

**Background:**

In the last few decades, several groups have observed that proteins expressed as inclusion bodies (IBs) in bacteria could still be biologically active when terminally fused to an appropriate aggregation-prone partner such as pyruvate oxidase from *Paenibacillus polymyxa* (PoxB). More recently, we have demonstrated that three amphipathic self-assembling peptides, an alpha helical peptide 18A, a beta-strand peptide ELK16, and a surfactant-like peptide L_6_KD, have properties that induce target proteins into active IBs. We have developed an efficient protein expression and purification approach for these active IBs by introducing a self-cleavable intein molecule.

**Results:**

In this study, the self-assembling peptide GFIL8 (GFILGFIL) with only hydrophobic residues was analyzed, and this peptide effectively induced the formation of cytoplasmic IBs in *Escherichia coli* when terminally attached to lipase A and amadoriase II. The protein aggregates in cells were confirmed by transmission electron microscopy analysis and retained ~50% of their specific activities relative to the native counterparts. We constructed an expression and separation coupled tag (ESCT) by incorporating an intein molecule, the *Mxe* GyrA intein. Soluble target proteins were successfully released from active IBs upon cleavage of the intein between the GFIL8 tag and the target protein, which was mediated by dithiothreitol. A variant of GFIL8, GFIL16 (GFILGFILGFILGFIL), improved the ESCT scheme by efficiently eliminating interference from the soluble intein-GFIL8 molecule. The yields of target proteins at the laboratory scale were 3.0–7.5 μg/mg wet cell pellet, which is comparable to the yields from similar ESCT constructs using 18A, ELK16, or the elastin-like peptide tag scheme.

**Conclusions:**

The all-hydrophobic self-assembling peptide GFIL8 induced the formation of active IBs in *E. coli* when terminally attached to target proteins. GFIL8 and its variant GFIL16 can act as a “pull-down” tag to produce purified soluble proteins with reasonable quantity and purity from active aggregates. Owing to the structural simplicity, strong hydrophobicity, and high aggregating efficiency, these peptides can be further explored for enzyme production and immobilization.

## Background

Overexpressed heterologous proteins in recombinant microbial hosts such as *Escherichia coli* often accumulate as insoluble inclusion bodies (IBs), which are generally considered to be biologically inactive and thus undesirable for protein expression and industrial applications [1–3]. Numerous efforts have been made to modulate or reduce the formation of IBs [4, 5]. However, over the last decade, the paradigm has completely changed. Several groups have observed that proteins deposited in IBs have biological activities. This was first reported by Worall in 1989 and 2 years later by Tokatlidis [6, 7]. The most universal and commonly used approaches to generate active IBs are to fuse a target protein to an aggregation-prone domain or protein sequence [8–12]. Several “pull-down” partners that drive proteins into active aggregates have been reported, including a virus capsid protein (VP1), a variant of a human β-amyloid peptide (Aβ(F19D)) [8], a mutant of the maltose-binding protein (MalE31) [9], a cellulose-binding domain from *Clostridium cellulovorans* (CBD_clos_) [10], pyruvate oxidase from *Paenibacillus polymyxa* (PoxB), [11] and the green fluorescent protein (GFP) [12]. More recently, a study has demonstrated that biologically active IBs for the GFP can be obtained through engineering the protein itself [13], but this approach seems to be strongly peptide or protein specific. Active IBs provide unique advantages compared with their soluble counterparts, such as easy separation and purification, greater stability and suitability as immobilized biocatalysis, bioassays, and biomaterials [14–16]. Thus, an increasing amount of attention has been drawn to this line of study [17, 18].

In our previous studies [19, 20], we found three self-assembling amphipathic peptides were able to serve as “pull-down” fusion tags to effectively induce several normally soluble proteins into cytoplasmic active IBs in *E. coli*, i.e., an alpha-helical octadecapeptide 18A (EWLKAFYEKVLEKLKELF), a beta-strand peptide ELK16 (LELELKLKLELELKLK) [19], and a small surfactant-like peptide L_6_KD (LLLLLLKD) [20]. Compared with other aggregating fusion partners, these peptides are much smaller in size and structurally simple, and generally have high “pull-down” efficiencies. Subsequently, we have developed a single-step protein purification approach by fusing a cleavable intein molecule between the target protein and the self-assembling peptide [21]. Thus, the target protein can be released into the soluble fraction by intein-mediated cleavage and easily obtained by centrifugation.

In this work, we tested a fourth type of peptide, an all-hydrophobic self-assembling peptide GFIL8 (GFILGFIL) that can induce active IBs when attached to the carboxyl termini of target proteins. This short peptide is inspired from the tetrapeptide (GFIL) [22], which can form gel-phase materials via self-assembly. This is the first study to use a short peptide composed of purely hydrophobic amino acids to induce the formation of active IBs and thereby demonstrates the potential of GFIL8 as a novel IB-inducing fusion tag in vivo. In addition, GFIL8 and its variant GFIL16 can also be successfully applied in the production and purification of proteins with the assistance of the intein molecule.

## Methods

### Plasmid construction

The construction of the plasmid encoding the fusion proteins LipA-GFIL8 was based on the plasmid pET30a-LipA-ELK16 [19]. The primers (LipA-For: 5′-ACGACGACATATGGCTGAACACAATCCAGT-3′, GFIL8-Rev: 5′-CCGCTCGAGTCACAGAATGAAACCCAGAATGAAACCCGGCGTCGGGGTTGG, the restriction sites *Nde*I and *Xho*I are underlined) were used to amplify the gene encoding LipA-GFIL8. The amplified LipA-GFIL8 gene was restriction digested with *Nde*I and *Xho*I, and inserted into the pET30a (+) (Novagen) vector to generate the pET30a-LipA-GFIL8 construct. The pET30a-AMA-GFIL8 plasmid was similarly constructed using primers AMA-For (5′-TTCTGGACATATGGCGGTAACCAAGTCATC-3′) and GFIL8-Rev. The construction of the plasmids encoding LipA (or AMA)-I-GFIL8 was based on the plasmids pET30a-LipA (or AMA)-I-ELK16 [21], using primers Intein-For (5′-TGATTGATGCACTAGTTGCCCTACCCGA-3′, the restriction site *Spe*I is underlined) and GFIL8-Rev.

### Expression and purification of IBs

*Escherichia coli* BL21 (DE3) (Novagen) cells were used for expressing the fusion proteins. The growth of the recombinant cells was carried out in Luria–Bertani (LB) medium supplemented with 50 mg/l kanamycin with shaking (250 rpm) at 37°C. Isopropyl β-d-1-thiogalactopyranoside with a final concentration of 0.2 mM was added to the cultures to initiate the expression of target proteins when the cell optical density (OD_600_) reached 0.4–0.6. Expression was continued for a further 6 h at 30°C. The strains were harvested by centrifugation at 6000×*g* for 10 min and cell pellets were stored at −80°C for further analysis. For LipA (or AMA)-GFIL8 fusion proteins, the cell pellets were resuspended in lysis buffer (50 mM Tris–HCl, 50 mM NaCl, 5% glycerol, pH 7.2) with a final concentration of 10 OD_600_/ml, and then thoroughly lysed by ultrasonication on ice. For fusion proteins incorporating the intein, the lysis buffer was replaced by buffer B1 (20 mM Tris–HCl, 500 mM NaCl, 1 mM EDTA, pH 8.5). The IBs were separated from the soluble fraction by centrifugation, then washed with lysis buffer twice, and finally resuspended in lysis buffer retaining the same volume. The amount of proteins in both fractions were densitometrically determined by denaturing polyacrylamide gel electrophoresis (SDS-PAGE, 12%) using bovine serum albumin (BSA) as the standard, followed by staining with Coomassie Brilliant Blue G-250, and calculated with Quantity One software (Bio-Rad Laboratories, Hercules, CA, USA).

### Enzymatic activity assay

The enzyme activities were measured in 96-well microplates with a SPECTRAMAX M2 microtiter reader (Molecular Device, Sunnyvale, CA, USA). The lipase activity [23] was measured by monitoring the formation of *p*-nitrophenol (pNP) following the *A*_405_ (ε, 18.7 cm^2^/μmol) at 37°C. Five microliters of diluted enzyme was added to 175 μl of the reaction buffer containing 50 mM sodium phosphate buffer (pH 8.0), 0.4 mM *p*-nitrophenyl palmitate, 0.2% sodium deoxycholate, and 0.1% gum arabic. The amadoriase activity [24] was carried out by monitoring the formation of a quinone dye following the *A*_555_ (ε, 39.2 cm^2^/μmol) in a peroxidase-coupling reaction at 37°C. The amadoriase assay was performed by adding 5 μl of the enzyme into 175 μl of the reaction mixture containing 100 mM potassium phosphate buffer (pH 8.0), 2.7 purpurogallin units of peroxidase, 0.45 mM 4-aminoantipyrine, 0.5 mM *N*-ethyl-*N*-(2-hydroxy-3-sulfopropyl)-m-toluidine (TOOS), and 5.0 mM d-fructosyl-glycine. One unit of lipase or amadoriase activity was defined as the amount of enzyme that produced 1 μmol pNP or 1 nmol H_2_O_2_ per min.

### Transmission electron microscopy analysis

A Hitachi H-7650B (Hitachi, Tokyo, Japan) transmission electron microscope (TEM) was used to analyze the morphology and intracellular location of protein aggregates. Cells were initially collected after expression for 6 h at 30°C, and fixed with 2.5% glutaraldehyde and 2% osmium tetraoxide. The cells were then embedded in epoxy resins after a graded-ethanol serial dehydration step. The embedded cells were sectioned into ultrathin slices, stained by uranyl acetate solution and lead citrate, and then observed with TEM at an accelerating voltage of 80 kV.

### Protein purification by intein-mediated cleavage

For fusion proteins incorporated with the intein, the IBs were washed with buffer B1 twice. The insoluble protein aggregates were then resuspended in buffer B3 (20 mM Tris–HCl, 500 mM NaCl, 1 mM EDTA, and 40 mM DTT, pH 8.5) to initiate the intein-mediated cleavage. The cleavage reactions were performed at 4°C for 24 h. The purified soluble proteins released from the IBs were obtained by centrifugation. The amount of proteins in all samples were determined by SDS-PAGE.

## Results

### Hydrophobic peptide GFIL8 induced the formation of active IBs

**Figure 1:**
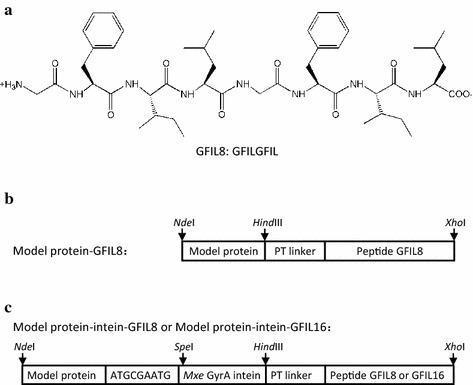
Schematics for hydrophobic peptide GFIL8 and fusion protein constructs. **a** Chemical structure of hydrophobic peptide GFIL8. **b** Genetic constructs of the model protein-GFIL8 fusion. **c** Genetic constructs of the model protein-intein-GFIL8 (or GFIL16) fusion. PT linker, PTPPTTPTPPTTPTPTP; model proteins, *Bacillus subtilis* lipase A (LipA) or *Aspergillus fumigatus* amadoriase II (AMA).

**Figure 2:**
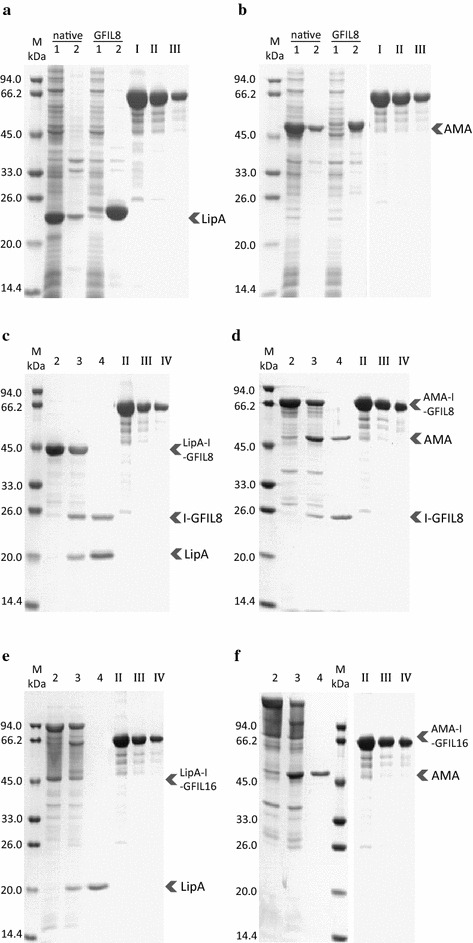
Fusion proteins expression and intein-mediated cleavage. **a** LipA-native and LipA-GFIL8. **b** AMA-native and AMA-GFIL8. **c** LipA-I-GFIL8. **d** AMA-I-GFIL8. **e** LipA-I-GFIL16. **f** AMA-I-GFIL16. For **a**–**f** *lane 1* soluble fraction of cell lysate; *lane 2* insoluble fraction of cell lysate; *lane 3* insoluble fraction of cleaved fusion protein; *lane 4* soluble fraction of cleaved fusion protein. *Lane I*, *II*, *III* and *IV*, bovine serum albumin (BSA) standards, at 6, 3, 1.5 and 0.75 μg/lane, respectively.

**Figure 3:**
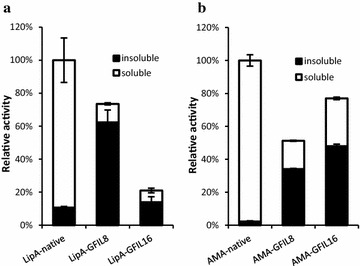
Distributions of enzymatic activities in the soluble and insoluble fractions of cells lysates. **a** LipA-native, LipA-GFIL8 and LipA-GFIL16. **b** AMA-native, AMA-GFIL8 and AMA-GFIL16. The activities were measured in triplicate with three independent expression clones and normalized to the total activities of the respective native enzyme extracted from a same amount of cells (OD_600_). Standard deviations are shown.

Unlike 18A, ELK16, and L_6_KD, the self-assembling tetrapeptide GFIL possesses neither polar nor electrically charged amino acids. The completely hydrophobic peptide GFIL has been reported to self-assemble into nanofibers with a cross-β structure at physiological pH [22]. Because the self-assembly interactions may be too weak for GFIL to induce proteins into aggregates, we doubled the sequence and constructed the fusion proteins in *E. coli* (Figure 1a, b). A rigid PT linker was used to fuse GFIL8 to the carboxyl termini of *Bacillus subtilis* lipase A (LipA, PDB code: 1I6W) and *Aspergillus fumigatus* amadoriase II (AMA, PDB code: 3DJD). The fusion proteins were expressed in *E. coli* at 30°C for 6 h using the inducer isopropyl β-d-1-thiogalactopyranoside (IPTG). Compared with native proteins, the terminal attachment of the GFIL8 fusion tag had negligible effect on cell growth and IBs were found following bacteriolysis. The lysates were then distributed into soluble and insoluble fractions by centrifugation and analyzed by sodium dodecyl sulfate-polyacrylamide gel electrophoresis (SDS-PAGE). Nearly 90% of the total fusion proteins were pulled down into the insoluble fraction, whereas for the native proteins the insoluble fraction contained only 20% of the target protein (Figure 2a, b). Both of the two model proteins in IBs were detected to be biologically active. For the LipA-GFIL8 fusion, the enzyme activity in the insoluble fraction accounted for 84.9% of the total activity, and for the AMA-GFIL8 fusion, the ratio was 66.5% (Figure 3). When the total activities of the two native enzymes were used as the corresponding benchmark, the IBs induced by GFIL8 retained 62.3% of LipA activity and 34.1% of AMA activity (Figure 3). The enzyme activities and relative specific activities are presented in detail in Table 1.

**Table 1:** Enzymatic activities of the fusion proteins expressed in *E. coli*

Enzymes	Activity (U/ml)^a^		Percent of activity in insoluble fractions (PDE)^b^	Percent of amount in insoluble fractions^c^	Specific activity (U/mg enzyme)^d^	Specific activity to the native enzyme in the studied phase (%)
	Soluble fractions	Insoluble fractions				
LipA-native	11.3 ± 1.7	1.3 ± 0.1	10.3	20.3	28.9 ± 4.4	100
LipA-GFIL8	1.4 ± 0.1	7.9 ± 1.0	84.9	88.6	12.5 ± 1.7	43.3
AMA-native	952.8 ± 34.0	22.5 ± 3.7	2.3	21.2	1591.7 ± 56.8	100
AMA-GFIL8	167.7 ± 2.8	332.5 ± 9.8	66.5	93.2	865.5 ± 91.4	54.4

^a^Cells were harvested 6 h after IPTG induction. The enzyme in the soluble fraction was extracted from cells with a final OD_600_ = 10. The insoluble fraction was taken from cells with a final OD_600_ = 10 by centrifugation and this pellet was re-suspended in lysis buffer to the same volume.

^b^Percentage activity in the insoluble fractions relative to the total activity in the cell lysate (soluble and insoluble fractions combined), also termed pull-down efficiency (PDE).

^c^Enzyme amounts were calculated by SDS-PAGE with serial concentrations of BSA as standards.

^d^The value for the native enzymes represents the enzyme in the soluble fractions. The value for the GFIL8 fusion represents the enzyme in protein aggregates.

**Figure 4:**
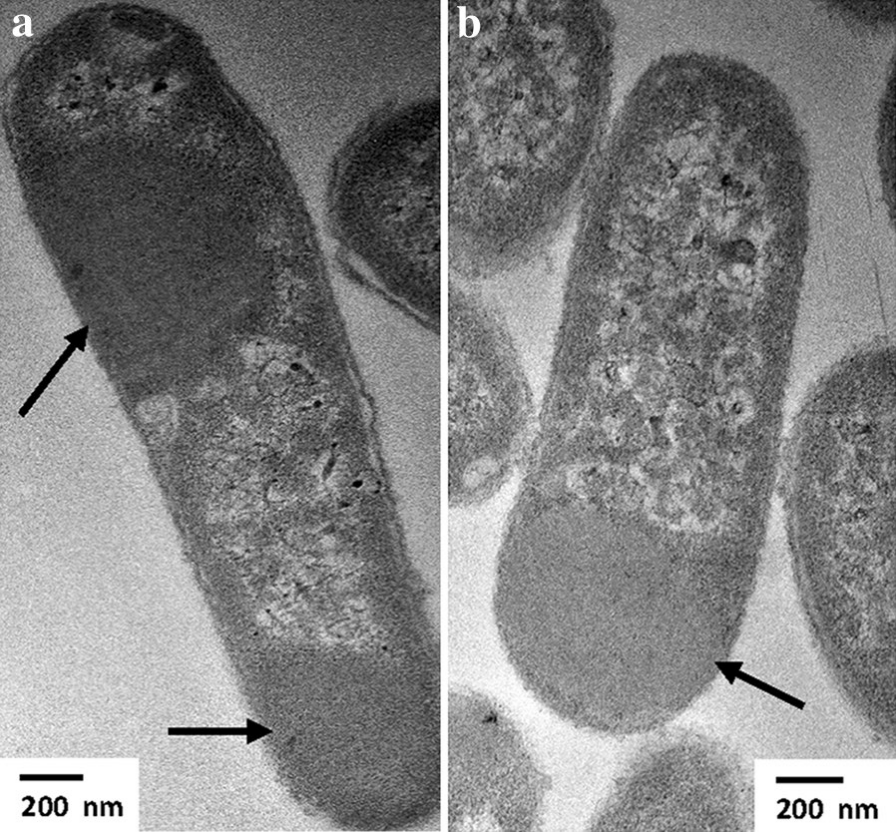
Intracellular localization of the GFIL8 fusion proteins in *E. coli*. **a**, **b** TEM microscopic images for LipA-GFIL8 and AMA-GFIL8, respectively. The *arrows* show the newly formed inclusion bodies in the recombinant cell. *Size bars* 200 nm.

To further study the intracellular locations of the GFIL8 fusion proteins, the recombinant cells were analyzed by transmission electron microscopy (TEM) to confirm the formation of IBs in vivo. As shown in Figure 4a, for cells expressing the LipA-GFIL8 fusion protein, a large proportion of the cytoplasm was occupied by a lump of protein aggregates (arrows). A similar distribution pattern was generally observed for AMA-GFIL8 (Figure 4b).

Naskar et al. [22] reported that another tetrapeptide (GAIL) could also self-assemble into hydrogels. Thus, as a comparison, a similarly extended peptide GAIL8 (GAILGAIL) was also tested in this work. However, <10% of GAIL8 fusion proteins could be pulled down into insoluble fractions (data not shown). This underscores the importance of hydrophobicity in inducing active protein aggregates for GFIL8.

### Intein-mediated protein production from active IBs

It would be useful to produce functional proteins if the target proteins from active IBs could be released into the soluble fraction. Thus, a commercialized intein, the *Mxe* GyrA intein [25] was incorporated between the model protein and the GFIL8 fusion tag (Figure 1c). This intein carries one mutation (Asn198Ala) that eliminates the C-terminal cleavage, and the N-terminal cleavage occurs with the addition of dithiothreitol (DTT). Three extra amino acid residues (MRM) were attached upstream of the intein to facilitate cleavage [26].

The fusion LipA-*Mxe* GyrA intein-GFIL8 (LipA-I-GFIL8) was first expressed as protein aggregates in *E. coli* (lane 2 in Figure 2c). Then, the aggregates were separated from the lysates and treated with 40 mM DTT at 4°C for 24 h to achieve intein-mediated cleavage. Both the insoluble and soluble fractions were then analyzed by SDS-PAGE (lanes 3 and 4 in Figure 2c, respectively). Protein quantification was performed by densitometry analysis using the software Quantity One (Bio-Rad Laboratories), and the data are presented in Table 2. Approximately 64.0% of the LipA-I-GFIL8 aggregates were successfully cleaved. The yield of soluble LipA after cleavage was 7.5 μg/mg wet cell pellet, accounting for 91.5% of the total cleaved LipA (the remaining LipA was entrapped in the aggregates). The recovery percentage (defined as the mass ratio of actually obtained soluble protein over the theoretical maximum yield assuming complete cleavage and release) of LipA was 58.6%. Similar aggregation and cleavage results were calculated for the AMA-I-GFIL8 fusion. The cleavage efficiency of AMA-I-GFIL8 aggregates was 64.5%. The yield of soluble AMA was 3.0 μg/mg wet cell pellet, or 20.6% in terms of the recovery percentage, indicating that a majority of the cleaved AMA (about 68.1%) was retained in the protein aggregates.

**Table 2:** Quantification of intein-mediated protein production

Product protein (molecular weight)	Aggregates^a^ (μg/mg wet cell pellet)	Quantity of purified protein^a^ (μg/mg wet cell pellet)	Specific activity (U/mg enzyme)	Cleavage efficiency^b^ (%)	Percent recovery^c^ (mass)
For GFIL8 fusion					
LipA (21 kDa)	27.1 ± 0.7	7.5 ± 0.7	34.6 ± 2.2	64.0 ± 2.7	58.6 ± 4.8
AMA (49 kDa)	21.4 ± 1.8	3.0 ± 0.3	1656.5 ± 48.9	64.5 ± 2.7	20.6 ± 0.9
For GFIL16 fusion					
LipA		4.7 ± 0.4	33.8 ± 0.5	49.0 ± 4.3	
AMA		4.3 ± 0.7	1567.7 ± 43.3	41.1 ± 5.5	

^a^Yield of protein from LB culture with wet cell weight of 2.66 ± 0.99 mg/ml.

^b^Cleavage efficiency was defined as the mass ratio of the amount of cleaved protein aggregate over that of the total aggregate before cleavage.

^c^Percent recovery was defined as the mass ratio of the amount of actually obtained protein released into the soluble fraction after cleavage over the theoretical maximum yield from the respective protein aggregate, assuming complete cleavage and release.

Although proteins expressed as IBs could be separated from intracellular protein impurities by centrifugation (lane 2 in Figure 2c, d), as seen for constructs using 18A, another band representing an intein-GFIL8 fragment (I-GFIL8) also appeared in the soluble fraction (lane 4 in Figure 2c, d). This suggests that the *Mxe* GyrA intein itself is difficult to be completely pulled down by GFIL8. Thus, I-GFIL8 fragments were partially soluble after DTT cleavage. To eliminate such disaggregation and yield pure target proteins, an attempt was made to double the length of GFIL8 peptide (named as GFIL16 with this sequence: GFILGFILGFILGFIL) to strengthen the self-assembly mediated by the hydrophobic effect. As expected, there was only one distinct band corresponding to LipA in the soluble fraction following cleavage, and similarly so for AMA-I-GFIL16, indicating that I-GFIL16 was almost insoluble (lane 4 in Figure 2e, f). The yield of soluble protein from GFIL16-induced aggregates was 4.7 μg/mg wet cell pellet for LipA and 4.3 μg/mg wet cell pellet for AMA (Table 2). For both LipA-I-GFIL16 and AMA-I-GFIL16 fusions, interestingly, the aggregates reflected in SDS-PAGE appeared as diffuse bands, and concentrated samples gave bands in the SDS-PAGE indicative of dimer formation (lane 2 in Figure 2e, f), which could not be denatured to monomers even by 6 mol/l guanidine hydrochloride or 8 mol/l urea (data not shown). We suspected that this observation arises because the GFIL16-induced aggregates are not completely solubilized by SDS. Because the aggregate amounts could not be estimated from the irregular bands in SDS-PAGE analysis, the cleavage efficiency was roughly estimated to be 49.0% for LipA and 41.1% for AMA.

Along this line, while GFIL8 fusions were found to slightly improve cell growth, GFIL16 fusions significantly reduced the OD_600_ at 7.5 h by 30–50% compared with the wild type cells with no plasmid. When the fusions with GFIL16 were also constructed and assayed for LipA and AMA, we found that LipA fused with GFIL16 showed a very low activity in both the soluble and insoluble fractions, while AMA-GFIL16 showed a higher activity than that of AMA-GFIL8 in both fractions (Figure 3). This result is quite similar with a previous construct where ELK16 was used as the pull-down tag [19]. In that case, we surmised that the ELK16-induced LipA aggregates were tightly packed, as a consequence the large substrate *p*-nitrophenyl palmitate (MW = 377.5) was difficult to enter the aggregates. GFIL16 likely has a similar effect. For AMA, the substrate d-fructosyl-glycine (MW = 237.1) is smaller, and no such effect was observed.

## Discussion

This study demonstrates that the C-terminally attached, hydrophobic peptide GFIL8 acts as a highly efficient “pull-down” tag to convert soluble proteins into active IBs. Such protein aggregates retain ~50% of the specific activities relative to the native soluble counterparts. The intracellular morphology of the protein aggregates was found to be similar to those reported in earlier studies using other aggregation-prone domains or peptides [27]. The PT linker between the peptide and the target protein being equal, peptide GFIL8 is only eight residues in length, the same as L_6_KD, and shorter than the peptides 18A (18 residues) and ELK16 (16 residues). The absence of polar or charged residues in GFIL8 indicates that aggregate formation depends completely on hydrophobic interactions. By replacing the Phe in GFIL8 with the hydrophobic residue Ala, peptide GAIL8 showed negligible protein aggregating properties. This proves that *π*–*π* stacking interactions between phenylalanine residues of peptide GFIL8 play a significant role in the aggregating process.

Because the protein aggregates are biologically active, this observation indicates that a large fraction of the proteins within the aggregates hold the active conformation. In our work, soluble target proteins have been successfully released from the aggregates using the *Mxe* GyrA intein system. The I-GFIL8 fragments upon cleavage are partially soluble and thus contaminate the target protein samples. By simply repeating the GFIL8 sequence, the GFIL16 peptide can associate more strongly, and efficiently render I-GFIL16 completely insoluble, and thus eliminate the presence of soluble I-GFIL8 fragments. Nearly no disaggregation occurred in I-GFIL16 precipitates even when treated with 6 mol/l guanidine hydrochloride or 8 mol/l urea, much as LipA-I-GFIL16 or AMA-I-GFIL16.

The yields of highly pure proteins released from IBs on the laboratory scale are in the range of 3.0–7.5 μg/mg wet cell pellet (Table 2). These yields are comparable to the yields from the 18A, ELK16, and the elastin-like peptide tag purification strategy (1.6–10.4 μg/mg wet cell pellet) [21, 28] and higher than those of the classical his-tag purification approach [29]. The specific activities of released proteins (Table 2) were rather comparable to the native counterparts (Table 1), which suggested that GFIL8- and GFIL16-induced aggregates did not interfere with the correct folding of the target proteins. Peptide GFIL8 and its variant GFIL16 have potential biotechnological applications on designing expression and purification coupled tags (ESCT) [21, 26], and producing active IBs for direct use as biocatalysts [14, 30]. Because the self-assembly of GFIL8 (or GFIL16) depends solely on hydrophobicity, we surmise that this tag can also be applied in the production of proteins under extreme pH conditions in vitro.

## Conclusions

The hydrophobic self-assembling peptide GFIL8 can be used as a novel IB-inducing fusion tag to convert soluble proteins into active aggregates in *E. coli*. Further studies revealed that the peptides GFIL8 and its variant GFIL16 can be successfully used in the production of proteins with reasonable quantity and purity via intein-mediated cleavage. Owing to the simplicity, strong hydrophobicity, and high aggregating efficiency of GFIL8 and GFIL16, these peptides represent significant potential to further explore this type of peptide design for applications in protein production, enzyme catalysis, and immobilization.

## References

[1] Georgiou G, Valax P (1996). Expression of correctly folded proteins in *Escherichia coli*. Curr Opin Chem Biol.

[2] Baneyx F, Mujacic M (2004). Recombinant protein folding and misfolding in *Escherichia coli*. Nat Biotechnol.

[3] de Marco A (2008). Minimal information: an urgent need to assess the functional reliability of recombinant proteins used in biological experiments. Microb Cell Fact.

[4] Martinez-Alonso M, Gonzalez-Montalban N, Garcia-Fruitos E, Villaverde A (2009). Learning about protein solubility from bacterial inclusion bodies. Microb Cell Fact.

[5] Vallejo LF, Rinas U (2004). Strategies for the recovery of active proteins through refolding of bacterial inclusion body proteins. Microb Cell Fact.

[6] Worrall DM, Goss NH (1989). The formation of biologically active beta-galactosidase inclusion bodies in *Escherichia coli*. Aust J Biotechnol.

[7] Tokatlidis K, Dhurjati P, Millet J, Beguin P, Aubert JP (1991). High activity of inclusion bodies formed in *Escherichia coli* overproducing *clostridium thermocellum* endoglucanase d. FEBS Lett.

[8] Garcia-Fruitos E, Gonzalez-Montalban N, Morell M, Vera A, Ferraz RM, Aris A (2005). Aggregation as bacterial inclusion bodies does not imply inactivation of enzymes and fluorescent proteins. Microb Cell Fact.

[9] Arie J-P, Miot M, Sassoon N, Betton JM (2006). Formation of active inclusion bodies in the periplasm of *Escherichia coli*. Mol Microbiol.

[10] Nahalka J, Nidetzky B (2007). Fusion to a pull-down domain: a novel approach of producing *Trigonopsis variabilis*d-amino acid oxidase as insoluble enzyme aggregates. Biotechnol Bioeng.

[11] Park SY, Park SH, Choi SK (2012). Active inclusion body formation using *Paenibacillus polymyxa* PoxB as a fusion partner in *Escherichia coli*. Anal Biochem.

[12] Huang Z, Zhang C, Chen S, Ye F, Xing XH (2013). Active inclusion bodies of acid phosphatase PhoC: aggregation induced by GFP fusion and activities modulated by linker flexibility. Microb Cell Fact.

[13] Raghunathan G, Munussami G, Moon H, Paik HJ, An SSA, Kim YS (2014). A variant of green fluorescent protein exclusively deposited to active intracellular inclusion bodies. Microb Cell Fact.

[14] Roessl U, Nahalka J, Nidetzky B (2010). Carrier-free immobilized enzymes for biocatalysis. Biotechnol Lett.

[15] Nahalka J, Mislovicova D, Kavcova H (2009). Targeting lectin activity into inclusion bodies for the characterisation of glycoproteins. Mol Biosyst.

[16] Garcia-Fruitos E, Rodriguez-Carmona E, Diez-Gil C, Ferraz RM, Vazquez E, Corchero JL (2009). Surface cell growth engineering assisted by a novel bacterial nanomaterial. Adv Mater.

[17] Garcia-Fruitos E (2010). Inclusion bodies: a new concept. Microb Cell Fact.

[18] Lotti M (2011). Bacterial inclusion bodies as active and dynamic protein ensembles. FEBS J.

[19] Wu W, Xing L, Zhou B, Lin Z (2011). Active protein aggregates induced by terminally attached self-assembling peptide ELK16 in *Escherichia coli*. Microb Cell Fact.

[20] Zhou B, Xing L, Wu W, Zhang XE, Lin Z (2012). Small surfactant-like peptides can drive soluble proteins into active aggregates. Microb Cell Fact.

[21] Xing L, Wu W, Zhou B, Lin Z (2011). Streamlined protein expression and purification using cleavable self-aggregating tags. Microb Cell Fact.

[22] Naskar J, Palui G, Banerjee A (2009). Tetrapeptide-based hydrogels: for encapsulation and slow release of an anticancer drug at physiological pH. J Phys Chem B.

[23] Winkler UK, Stuckmann M (1979). Glycogen, hyaluronate, and some other polysaccharides greatly enhance the formation of exolipase by *Serratia marcescens*. J Bacteriol.

[24] Zheng J, Guan H, Xu L, Yang R, Lin Z (2010). Engineered amadoriase II exhibiting expanded substrate range. Appl Microbiol Biotechnol.

[25] Telenti A, Southworth M, Alcaide F, Daugelat S, Jacobs WR, Perler FB (1997). The *Mycobacterium xenopi* GyrA protein splicing element: characterization of a minimal. J Bacteriol.

[26] Ge X, Yang DSC, Trabbic-Carlson K, Kim B, Chilkoti A, Filipe CDM (2005). Self-cleavable stimulus responsive tags for protein purification without chromatography. J Am Chem Soc.

[27] Williams DC, Vanfrank RM, Muth WL, Burnett JP (1982). Cytoplasmic inclusion-bodies in *Escherichia*-*coli* producing biosynthetic human insulin proteins. Science.

[28] Banki MR, Feng LA, Wood DW (2005). Simple bioseparations using self-cleaving elastin-like polypeptide tags. Nat Methods.

[29] Banki MR, Wood DW (2005). Inteins and affinity resin substitutes for protein purification and scale up. Microb Cell Fact.

[30] Sheldon RA (2011). Cross-linked enzyme aggregates as industrial biocatalysts. Org Process Res Dev.

